# Association of haemato-biochemical indices and blood composite ratios with microfilaridermia in Onchocerciasis patients

**DOI:** 10.1186/s12879-024-09278-0

**Published:** 2024-04-08

**Authors:** Linda Batsa Debrah, Charles Gyasi, Monica Ahiadorme, Abu Abudu Rahamani, Vera Serwaa Opoku, Prince Obeng, Jubin Osei-Mensah, Michael Agyemang Obeng, Derrick Adu Mensah, Alexander Yaw Debrah

**Affiliations:** 1 Department of Clinical Microbiology, School of Medicine and Dentistry, Kwame Nkrumah University of Science and Technology, Kumasi, Ghana; 2 Kumasi Centre for Collaborative Research in Tropical Medicine, Kwame Nkrumah University of Science and Technology, Kumasi, Ghana; 3 Department of Pathobiology, School of Veterinary Medicine, Kwame Nkrumah University of Science and Technology, Kumasi, Ghana; 4 Department of Medical Diagnostics, Faculty of Allied Health Sciences, Kwame Nkrumah University of Science and Technology, Kumasi, Ghana

**Keywords:** Onchocerciasis, Onchocercomata, Microfilaria, Haemato-biochemical indices, Blood composite ratios, Eosinophil, Eosinophil-to-monocyte ratio, Eosinophil-to-Neutrophil ratio.

## Abstract

**Background:**

Onchocerciasis causes chronic systemic inflammation. Several studies have used markers such as haemato-biochemical indices to predict the occurrence of systemic inflammation. This study assessed the variability and predictability of haemato-biochemical indices and blood composite ratios (BCRs) in microfilariae positive (MF+) and microfilariae negative (MF-) subgroups of onchocercomata participants.

**Methods:**

One hundred and five (105) MF + and 34 MF- participants were retrospectively recruited into the study. Screening for the presence of *O. volvulus* microfilariae was done from skin snips taken from the left and right iliac crests of participants using established and approved protocols. Haematological and biochemical indices were measured using standard laboratory automated analyzers. Blood composite ratios (BCRs) were calculated as ratios of the absolute parameters involved.

**Results:**

A significantly increased total WBC, absolute eosinophil, eosinophil percent and absolute basophil were observed in the MF + participants compared to MF- participants. Reduced gamma-glutamyl transferase (GGT) with increased estimated glomerular filtration rate (eGFR) was significantly associated with MF + participants compared to MF- participants. BCRs were significantly higher for eosinophil-to-neutrophil ratio (ENR), eosinophil-to-monocyte ratio (EMR), eosinophil-to-basophil ratio (EBR) and eosinophil-to-lymphocyte ratio (ELR) in MF + participants compared to MF- participants. After multivariate adjustment, onchocercomata participants with increased eosinophil counts (aOR = 13.86, 95% CI [2.07–92.90], *p* = 0.007), ENR x10 (aOR = 1.42, 95% CI [1.05–1.93], *p* = 0.025), EMR (aOR = 2.64, 95% CI [1.25–5.60], *p* = 0.011), EBR (aOR = 1.07, 95% CI [1.01–1.10], *p* = 0.020) and ELR x10 (aOR = 1.69, 95% CI [1.14–2.51], *p* = 0.009) were more likely to have microfilaridermia.

**Conclusions:**

Elevated eosinophil counts with higher ENR, EMR, EBR and ELR levels are significantly associated with microfilaridermia in onchocercomata participants. Combining BCRs with eosinophil count significantly led to an improvement in the conventional model for predicting microfilaridermia.

**Supplementary Information:**

The online version contains supplementary material available at 10.1186/s12879-024-09278-0.

## Background

Onchocerciasis, a chronic helminth infection, is one of the leading causes of morbidity and mortality worldwide [1, 2]. The infection is caused by a filarial nematode *Onchocerca volvulus* and transmitted by blackfly vectors [3]. In humans, onchocerciasis can lead to severe dermal and ocular pathologies often as a result of an acute inflammatory response to dead microfilariae (MF) [4]. Community-directed treatment with ivermectin (CDTI) remains the World Health Organization (WHO) strategy for achieving high therapeutic coverage, and elimination of the infection with a global target of 12 (31%) endemic countries verified for having interrupted transmission by 2030 [5]. CDTI approach has improved treatment coverage and subsequently broke transmission cycles― radically altering the consequences of *O. volvulus* infection [6, 7]. However, recent reports show that CDTI alone is unlikely to eliminate onchocerciasis in areas where fear of ivermectin (IVM) adverse events and death are detrimentally anchored in the population [1, 8]. Alternative strategies are therefore needed for onchocerciasis elimination [8, 9], and research on diagnostic factors in this group is of special interest.

The clinical spectrum of human onchocerciasis manifests as two polar forms among infected participants who reside in areas that have undergone none or only a few rounds of mass drug administration (MDA) [10]. The hyporesponsive form, characterized by participants with palpable onchocercomas (nodules) but no strong pathology amidst high MF skin loads, is more common than the hyperresponsive form [10] which manifests as sowda with few or no skin MF. In addition to these clinical manifestations, a subgroup of the hyporesponsive form has been reported in hyperendemic areas that have undergone multiple rounds of ivermectin (IVM) [11, 12]. This subgroup, believed to stem from the effects of repeated preventive chemotherapy with IVM, has palpable nodules with adult worms but are a-microfilaridermic and manifest little or no dermal/skin and ocular pathology(-ies) [13]. To date, there remains a clinical dearth of understanding between this population of a-microfilaridermic (MF-) and hyporesponsive forms (MF+).

A strong inflammatory response through the activation of inflammatory cells is characteristic of helminth infection [14]. Onchocerciasis is known to activate both the innate and adaptive immune system cells in the host [15, 16]. *O. volvulus* infection is characterized by type 2 immune response with changes in lymphocytes, eosinophils, basophils, neutrophils and mast cell distributions [17]. Several studies have shown that the vast majority of incoming L3 are killed by innate immune cells [14, 17, 18]. Also, evidence from other reports shows that granulocytes are key mediators of microfilariae killing following antihelminthic treatment [13, 14].

High neutrophil levels after multiple rounds of MDA have been implicated in a-microfilaridermias [13, 19]. The neutrophils in these participants correlated negatively with eosinophils [13]. Also, high eosinophil levels with low neutrophil and basophil levels have been implicated in MF + participants [13, 20]. Thus, suggesting a potential interaction among granulocytes in onchocerciasis infection. Further to this, without concomitant neutrophil involvement, high eosinophil counts alone may not predict distinction among onchocercomata participants with or without skin MF [16, 19].

Blood composite ratios (BCRs) of inflammatory cells are novel and stable biomarkers of vast clinical relevance in inflammatory diseases, mortality and prognosis [21–23]. A growing number of studies suggest that BCRs may serve as an independent discriminatory predictor of chronic diseases [24–26]. Thus, BCRs such as eosinophil-to-neutrophil ratio (ENR), eosinophil-to-basophil ratio (EBR), eosinophil-to-monocyte ratio (EMR), neutrophil-to-lymphocyte ratio (NLR) and eosinophil-to-lymphocyte ratio (ELR) may be stable biomarkers in onchocerciasis disease. However, the variability and predictability of these ratios have not been previously elucidated in MF + and MF- onchocerciasis participants. To resolve this, we examined the haemato-biochemical profile and selected BCRs among MF + and MF- participants in the Sefwi Akontombra district of Ghana.

## Materials and methods

### Ethical consideration

Ethical approval of the study was obtained from the Committee on Human Research Publication and Ethics (CHRPE) of the School of Medicine and Dentistry of the Kwame Nkrumah University of Science and Technology (KNUST), Kumasi, Ghana. Community leaders were consulted at the beginning of the study for their support and permission, and written approval was sought from the Western North Regional and the Sefwi Akontombra District Health Directorates. Informed consent was obtained from all participants and/or their legal guardians either by thumb printing or signing. This study was part of other larger studies titled: “The efficacy of rifampicin 35 mg/kg/d plus albendazole 400 mg/d given for 7 or 14 days against onchocerciasis– a randomized, controlled, parallel-group, open-label, phase II pilot trial” with registration number PACTR202009704006025, and “Prevalence assessment for onchocerciasis in some selected districts in Ghana”. The ethical approvals received for these studies were CHRPE/AP/359/20 and CHRPE/AP/492/20.

### Study population

A total of 139 participants, made up of 105 MF + and 34 MF- participants (males and females) aged between 18 to 68 years were retrospectively recruited from November 2020 to April 2021. Recruitment of the *O. volvulus* infected participants was done from 25 communities located adjacent to and along the Tano river in the Sefwi Akontombra district of Ghana. The Sefwi Akontombra district lies between latitudes 6^o^ N and 6^o^ 30’ N and longitudes 2^o^ 45’ W and 2^o^ 15’ W in the north-eastern part of the Western North Region [27]. It is characterized by moderate to heavy rainfall of between 1,524 mm and 1,780 mm per annum, with the peak in September-October. The communities within the districts are intersected by the Tano River and other major tributaries like Suhein, Kunuma, Sui, and the Yoyo [27]. Agricultural activities primarily along the Tano river play a major role in the district’s economy through the production of cocoa, palm trees, and other food crops [27].

### Data collection

**Fig. 1:**
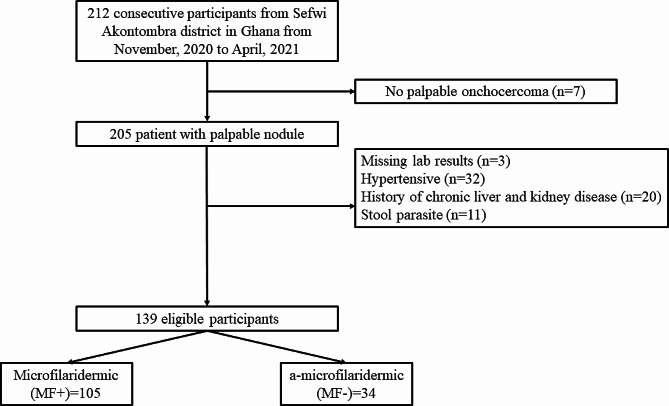
Flowchart of participant inclusion in the study

Demographic characteristics, clinical data, IVM intake status and laboratory findings for each participant were extracted from electronic records (REDCap). Electronic data were doubled entered by two independent data entry persons and were validated with original paper case report forms (pCRF). Participants were assured of the safety and confidentiality of their responses and laboratory data. Participants with a history of chronic diseases, haematological disorders, pregnancy, non-steroidal anti-inflammatory drug (NSAID) usage, recent antimalarial drug intake, incomplete or no laboratory results, intestinal helminths and other parasites, history of smoking and alcoholism were excluded from the study. Figure 1. shows the participant enrollment flowchart of the study. Participants were classified as MF + and MF- as previously reported [13, 20].

### MDA rounds/intake assessment

MDA has been implemented by the Ministry of Health in Ghana through the district’s disease control office for over 20 years in the district with considerable compliance. Therefore, at the time of sampling, most of the participants self-reported having taken part in the MDA programme from the district. The self-reported intake and participation in MDA programmes were used to assess individual IVM intake for each participant. To prevent MDA intake/treatment rounds recall bias, responses from participants were re-checked/cross-checked from the treatment records with the community health volunteers in the communities who had the MDA records for every household. This was done before the numbers were used in any analyses.

### Distance to the nearest river

Distances to the nearest river were measured using eTrex 10 (Garmin International, Inc., USA). Briefly, the coordinates of the communities were acquired using the GPS function of the eTrex 10 and referenced to the nearest river around the communities. The distance from a focal point in the community to the nearest river was then measured and recorded in kilometres (km).

### Parasitological and laboratory assessment

Participants with *O. volvulus* infection who had at least one palpable nodule were recruited for the study [28]. Screening for the presence of skin microfilariae (MF/mg of skin) was done as previously reported [13]. Infection with Plasmodium parasite(s), intestinal helminths and other parasites (protozoans and flagellates) ― were diagnosed using standard laboratory methods. Venous blood was collected by venipuncture from the median cubital superficial vein of the upper limb by sterile disposable hypodermic vacutainer needles into tubes with ethylenediaminetetraacetic acid (EDTA) as an anticoagulant (for haematological analytes) and gel clot activators (for biochemistry analytes). Haematological indices were then measured using a standard laboratory automated analyzer (Sysmex XN 350, USA). Serum alanine transaminase (ALT), aspartate transaminase (AST), gamma-glutamyl transferase (GGT) and creatinine were measured using an automated biochemical analyzer (Selectra ProS ELITech Clinical Chemistry, Germany) according to the manufacturer’s instructions. The calculation of eGFR was based on the formulae proposed by Levey et al. [29]. Blood Composite ratios were calculated as a ratio of the absolute parameters involved. Early morning stool samples from participants were collected and analyzed using wet mount and Kato-Katz techniques. In the Kato-Katz method, counting and recording of stool parasites were carried out according to the manufacturer’s instructions by experienced laboratory scientists.

### Statistical analysis

Statistical analysis was done using GraphPad Prism version 8.4.3 (GraphPad Software, Inc., San Diego, CA) and SPSS Statistics 25.0 software (SPSS Inc., Chicago, IL, USA). Continuous variables were expressed as median and interquartile ranges whereas categorical variables were expressed as frequencies and percentages. Comparisons between continuous variables were done using the Mann-Whitney U-test. The chi-square test and Fisher’s exact tests were performed for categorical variables where appropriate. The receiver operating characteristics (ROC) curve analyses were done to assess the predictability of microfilaridermia by the BCRs. Test for multicollinearity were conducted to identify and address high correlations between predictor variables, enhance model stability and improve interpretability. Predictors with Variable Inflation Factor (VIF) greater than 10 were excluded from further analysis. Univariate and multivariate logistic regression analyses were done to determine the association between BCRs and demographic characteristics. Furthermore, C-statistics were done to assess the incremental predictability of blood cell ratios. To optimize BCRs for clinical practice, their cut-off values with their specificity, sensitivity, negative predictive values (NPV) and positive predictive values (PPV) were assessed. Statistical significance was set at *p* < 0.05.

## Results

### Baseline characteristics of study participants

**Fig. 2:**
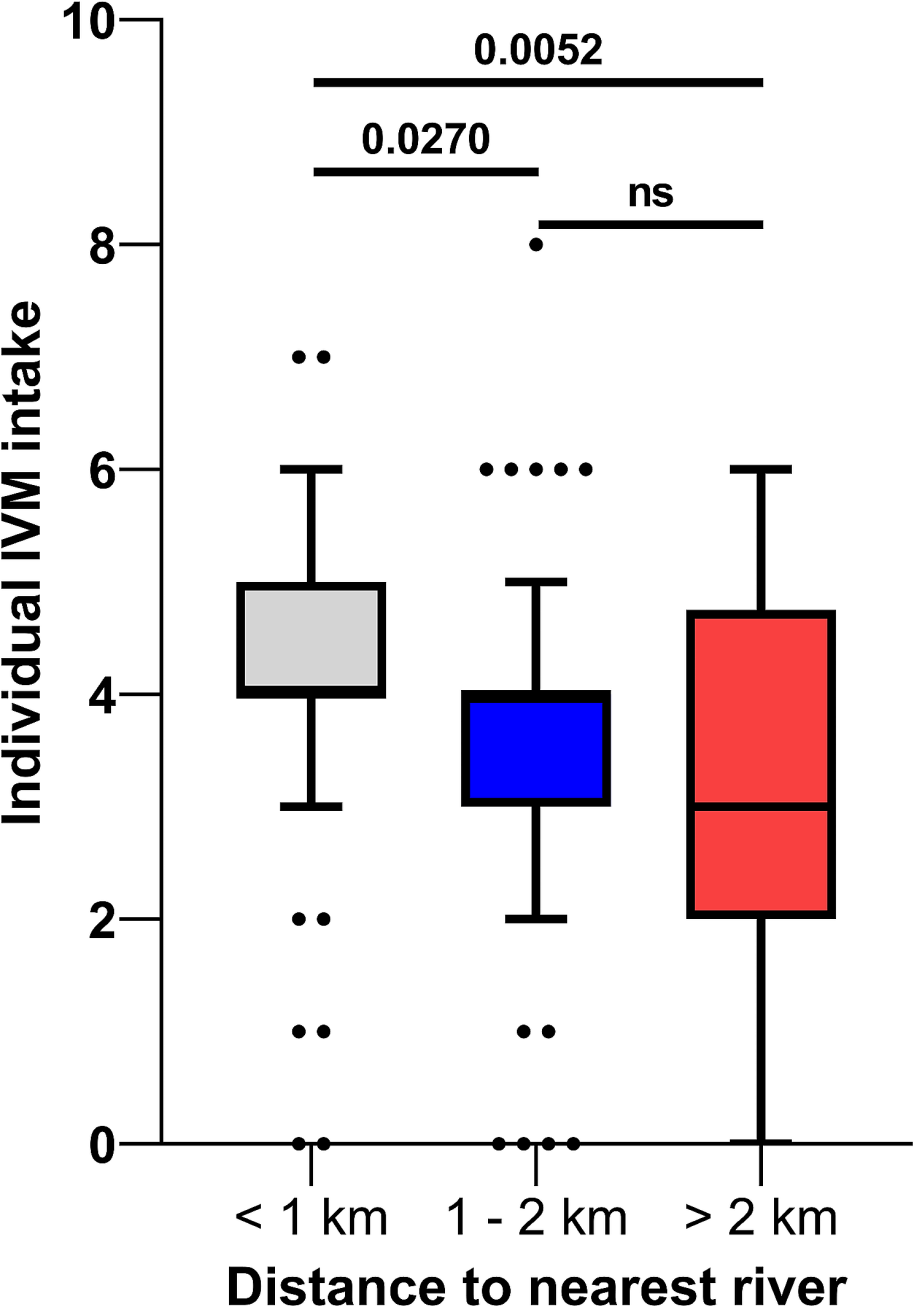
Distribution of ivermectin intake among study participants by distance to the nearest river

Among all enrolled onchocercomata participants, 105 were microfilaria-positive (MF+) and 34 were microfilaria-negative (MF-). MF + participants were younger, having a higher proportion of males and farmers than MF- participants (Table 1). The median years lived in endemic area for MF + participants were significantly lower (25.0 [15.0–30.0] vs. 30.0 [22.0–35.0], *p* = 0.007) than MF- participants (Table 1). A higher percentage of the MF- participants lived 1–2 km from the nearest river (58.8% vs. 38.1%, *p* = 0.046) compared to MF + participants (Table 1). Participants who lived < 1 km (4.0 [4.0–5.0]) from the nearest river had significantly taken higher rounds of MDA compared to those who either lived 1–2 km (4.0 [3.0–4.0]) or > 2 km (3.0 [2.0–4.8]) from the nearest river (Fig. 2).

A *p*-value less than 0.05 was considered statistically significant. ns; not significant, km; kilometre. Distance to the nearest river was measured as a community reference point to the nearest river using eTrex10 (Garmin International, Inc., USA).

**Table 1:** Baseline socio-demographic characteristics of study participants

Variable	N	a-Microfilaridermic (*n* = 34)	N	Microfilaridermic (*n* = 105)	*p*-value
Age (years)	34	41.5 [34.5–45.0]	105	37.0 [30.0–44.0]	0.070^¥^
Year in an endemic area (years)	34	30.0 [22.0–35.0]	105	25.0 [15.0–30.0]	**0.007** ^**¥**^
Gender (male, n.%)	34	22 [64.7]	105	70 [66.7]	0.834^a^
Distance to the nearest river (n.%)					
< 1.00 km	34	12 [35.3]	105	43 [41.0]	**0.046** ^**a**^
1.00–2.00 km		20 [58.8]		40 [38.1]	
> 2.00 km		2 [5.9]		22 [21.0]	
Occupation	34		105		**0.001** ^**b**^
Farmer		23 [67.7]		89 [84.8]	
Trader		8 [23.5]		3 [2.8]	
Others		3 [8.8]		13 [12.4]	
Has taken IVM before, n (%)	34	34 [100.0]	105	95 [90.5]	0.062^b^
Individual IVM intake	34	4.0 [3.75–5.0]	105	4.0 [3.0–5.0]	0.056^¥^
total nodule sites	34	1.0 [1.0–2.0]	105	2.0 [1.0–2.0]	0.089^¥^
total nodule counts	34	1.0 [1.0–2.3]	105	2.0 [1.0–3.0]	0.169^¥^
Mf skin load (Mf/mg)	34	0.00 [0.00–0.00]	105	0.62 [0.25–2.00]	**< 0.001** ^**¥**^

^*a*^: *chi-square test;*^*b*^: *Fishers’ exact test;*^*¥*^: *Mann-Whitney U-test analysis; km: kilometres; Mf/mg: microfilariae per milligram of skin weight*

### MF + participants exhibit disproportionate abnormalities in haematological and biochemical indices

Table 2 shows the abnormalities in haematological and biochemical indices of MF + and MF- participants based on reference ranges set by the manufacturers of the analyzers. MF + participants showed disproportionate abnormalities in haematological indices for RBC (6.7%), absolute eosinophil (51.4%), eosinophil percent (58.1%), absolute lymphocyte (6.7%), lymphocyte percent (18.1%), absolute monocyte (9.5%), monocyte percent (5.7%) and basophil percent (2.9%). Biochemical indices such as ALT (5.7%), AST (9.5%), GGT (7.6%), creatinine (2.9%) and eGFR (37.1%) were also disproportionately abnormal in MF + participants. Disproportionate abnormalities in RBC (8.8%), absolute eosinophil (14.7%), eosinophil percent (29.4%), absolute lymphocyte (2.9%), lymphocyte percent (26.5%), monocyte percent (2.9%), ALT (11.8%), AST (5.9%), GGT (11.8%), creatinine (8.8%) and eGFR (50.0%) were also observed in MF- participants.

**Table 2:** Haematological and biochemical ranges and abnormalities of study participants

Variable	unit	Classification	a-Microfilaridermics (MF-, *n* = 34)	Microfilaridermics (MF+, *n* = 105)	Total n (%)	Reference range
RBC	x10^6^/L	Normal	31 (91.2)	98 (93.3)	129 (92.8)	2.50–5.50
		Abnormal	3 (8.8)	7 (6.7)	10 (7.2)	
HGB	g/dL	Normal	34 (100.0)	105 (100.0)	139 (100.0)	8.0–17.0
		Abnormal	0 (0.0)	0 (0.0)	0 (0.0)	
HCT	%	Normal	34 (100.0)	105 (100.0)	139 (100.0)	26.0–50.0
		Abnormal	0 (0.0)	0 (0.0)	0 (0.0)	
PLT	x10^3^/L	Normal	33 (97.1)	104 (99.0)	137 (98.6)	50–400
		Abnormal	1 (2.9)	1 (1.0)	2 (1.4)	
Total WBC	x10^9^/L	Normal	34 (100.0)	104 (99.0)	138 (99.3)	3.00–15.00
		Abnormal	0 (0.0)	1 (1.0)	1 (0.7)	
Neutrophil Abs	x10^9^/L	Normal	34 (100.0)	104 (99.0)	138 (99.3)	1.50–7.00
		Abnormal	0 (0.0)	1 (1.0)	1 (0.7)	
Lymphocyte Abs	x10^9^/L	Normal	33 (97.1)	98 (93.3)	131 (94.2)	1.00–3.70
		Abnormal	1 (2.9)	7 (6.7)	8 (5.8)	
Monocyte Abs	x10^9^/L	Normal	34 (100.0)	95 (90.5)	129 (92.8)	0.00–0.70
		Abnormal	0 (0.0)	10 (9.5)	10 (7.2)	
Eosinophil Abs	x10^9^/L	Normal	29 (85.3)	51 (48.6)	80 (57.6)	0.00–0.40
		Abnormal	5 (14.7)	54 (51.4)	59 (42.4)	
Basophil Abs	x10^9^/L	Normal	34 (100.0)	105 (100.0)	139 (100.0)	0.00–0.10
		Abnormal	0 (0.0)	0 (0.0)	0 (0.0)	
Neutrophil %	%	Normal	34 (100.0)	104 (99.0)	138 (99.3)	37.0–72.0
		Abnormal	0 (0.0)	1 (1.0)	1 (0.7)	
Lymphocyte %	%	Normal	25 (73.5)	86 (81.9)	111 (79.9)	20.0–50.0
		Abnormal	9 (26.5)	19 (18.1)	28 (20.1)	
Monocyte %	%	Normal	33 (97.1)	99 (94.3)	132 (95.0)	0.0–14.0
		Abnormal	1 (2.9)	6 (5.7)	7 (5.0)	
Eosinophil %	%	Normal	24 (70.6)	44 (41.9)	68 (48.9)	0.0 − 6.0
		Abnormal	10 (29.4)	61 (58.1)	71 (51.1)	
Basophil %	%	Normal	34 (100.0)	102 (97.1)	136 (97.8)	0.0–1.0
		Abnormal	0 (0.0)	3 (2.9)	3 (2.2)	
ALT	U/L	Normal	30 (88.2)	99 (94.3)	129 (92.8)	0.0–40.0
		Abnormal	4 (11.8)	6 (5.7)	10 (7.2)	
AST	U/L	Normal	32 (94.1)	95 (90.5)	127 (91.4)	0.0–40.0
		Abnormal	2 (5.9)	10 (9.5)	12 (8.6)	
GGT	U/L	Normal	30 (88.2)	97 (92.4)	127 (91.4)	0.0–55.0
		Abnormal	4 (11.8)	8 (7.6)	12 (8.6)	
Creatinine	µmol/L	Normal	31 (91.2)	102 (97.1)	133 (95.7)	53.0–124.0
		Abnormal	3 (8.8)	3 (2.9)	6 (4.3)	
eGFR	mL/min/1.73m2	Normal	17 (50.0)	66 (62.9)	83 (59.7)	> 90.00
		Abnormal	17 (50.0)	39 (37.1)	56 (40.3)	

RBC; red blood cell count, HGB; haemoglobin concentration, PLT; platelet count, WBC; white blood cell count, ALT; alanine transaminase, AST; aspartate transaminase, GGT; gamma-glutamyl transferase, eGFR; estimated glomerular filtration rate

### MF + participants show significant variabilities in haematological and biochemical indices

MF + participants had significantly higher Total WBC (5.39 [4.78–6.60] vs. 4.71 [3.97–5.50], *p* = 0.001), absolute eosinophil (0.42 [0.19–0.84] vs. 0.14 [0.08–0.36], *p* < 0.0001), absolute basophil (0.03 [0.02–0.04] vs. 0.02 [0.01–0.03], *p* = 0.001) and eosinophil percent (8.0 [3.8–14.1] vs. 3.2 [1.9–7.5], *p* < 0.0001) with reduced GGT (25.3 [20.1–32.8] vs. 33.4 [21.9–39.6], *p* = 0.013) compared to MF- participants (Table 3). Strikingly, MF- participants had significantly lower eGFR levels (89.7 [82.8–94.8] vs. 94.8 [84.4–107.5], *p* = 0.040) compared to MF + participants (Table 3). However, RBC, HGB, PLT, absolute neutrophil, absolute lymphocyte, absolute monocyte, neutrophil percent, lymphocyte percent, basophil percent, monocyte percent, ALT, AST, and creatinine did not differ significantly (*p* > 0.05) between MF + and MF- participants (Table 3).

**Table 3:** Biochemical and Haematological Variabilities among Study Participants

Variable	unit	N	a-Microfilaridermic (*n* = 34)	N	Microfilaridermic (*n* = 105)	*p*-value
			median (IQR)		median (IQR)	
RBC	x10^9^/L	34	4.73 [4.24–5.24]	105	4.57 [4.20–4.95]	0.209
HGB	g/dL	34	13.5 [12.1–14.6]	105	13.1 [12.1–14.2]	0.390
HCT	%	34	41.2 [38.2–43.6]	105	40.1 [37.1–42.8]	0.253
PLT	x10^9^/L	34	196 [123–246]	105	200 [161–247]	0.343
Total WBC	x10^9^/L	34	4.71 [3.97–5.50]	105	5.39 [4.78–6.60]	**0.001**
Neutrophil Abs	x10^9^/L	34	1.78 [1.54–2.54]	105	1.99 [1.46–2.69]	0.602
Lymphocyte Abs	x10^9^/L	34	2.07 [1.79–2.44]	105	2.28 [1.85–2.85]	0.069
Monocyte Abs	x10^9^/L	34	0.43 [0.32–0.53]	105	0.47 [0.35–0.59]	0.110
Eosinophil Abs	x10^9^/L	34	0.14 [0.08–0.36]	105	0.42 [0.19–0.84]	**< 0.0001**
Basophil Abs	x10^9^/L	34	0.02 [0.01–0.03]	105	0.03 [0.02–0.04]	**0.001**
Neutrophil %	%	34	40.4 [35.6–48.4]	105	37 [31.2–44.7]	0.055
Lymphocyte %	%	34	44.9 [38.9–53.6]	105	42.2 [36.3–49.0]	0.198
Monocyte %	%	34	9.0 [7.7–9.8]	105	8.0 [6.5–10.5]	0.216
Eosinophil %	%	34	3.2 [1.9–7.5]	105	8.0 [3.8–14.1]	**< 0.0001**
Basophil %	%	34	0.4 [0.3–0.6]	105	0.5 [0.4–0.7]	0.057
ALT	U/L	34	24.0 [20.1–29.6]	105	22.4 [17.5–30.2]	0.513
AST	U/L	34	22.3 [18.5–29.0]	105	23.0 [17.8–28.5]	0.745
GGT	U/L	34	33.4 [21.9–39.6]	105	25.3 [20.1–32.8]	**0.013**
Creatinine	µmol/L	34	95.0 [79.0–103.0]	105	91.0 [79.5–101.0]	0.289
eGFR	mL/min/1.73m^2^	34	89.7 [82.8–94.8]	105	94.8 [84.4–107.5]	**0.040**

RBC = red blood cell; HGB = haemoglobin concentration; HCT = haematocrit; PLT = platelet; WBC = white blood cell; abs = absolute; ALT = alanine transaminase; AST = aspartate transaminase; GGT = gamma-glutamyl transferase; eGFR = estimated glomerular filtration; IQR = inter-quartile range. Mann-Whitney U-test was used for all analyses. *p*-value < 0.05 was considered statistically significant

### Increased blood composite ratios are significantly associated with MF + participants

**Fig. 3:**
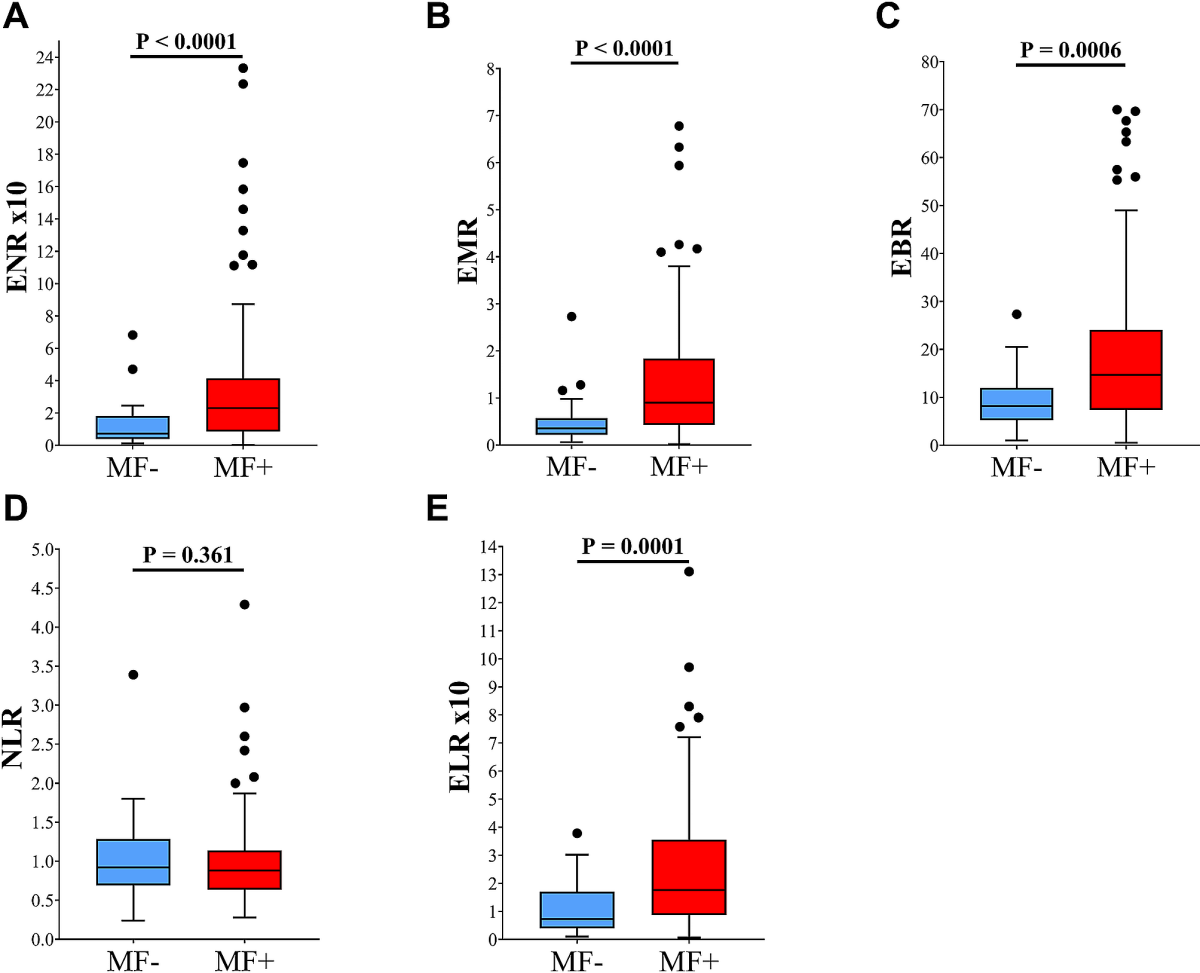
Variabilities in blood composite ratios among study participants ENR: eosinophil-to-neutrophil ratio; EBR: eosinophil-to-basophil ratio; EMR: eosinophil-to-monocyte ratio; NLR: neutrophil-to-lymphocyte ratio; ELR: eosinophil-to-lymphocyte ratio

Blood composite ratios (BCR) were significantly higher for ENR x10 (2.30 [0.87–4.14] vs. 0.73 [0.40–1.81], *p* < 0.0001), EMR (0.90 [0.43–1.84] vs. 0.36 [0.22–0.57], *p* < 0.0001), EBR (14.67 [7.38–24.07] vs. 8.20 [5.25–12.00], *p* = 0.0006) and ELR x10 (1.76 [0.87– 3.55] vs. 0.72 [0.40–1.70], *p* = 0.0001) in MF + participants compared to MF- participants (Fig. 3). Although MF + participants showed a lower NLR (0.88 [0.64–1.14] vs. 0.92 [0.69–1.29]) compared to MF- participants, no statistically significant difference was observed (*p* = 0.361) (Fig. 3).

### BCRs correlate positively with eosinophil but negatively with neutrophil levels in MF + participants

**Fig. 4:**
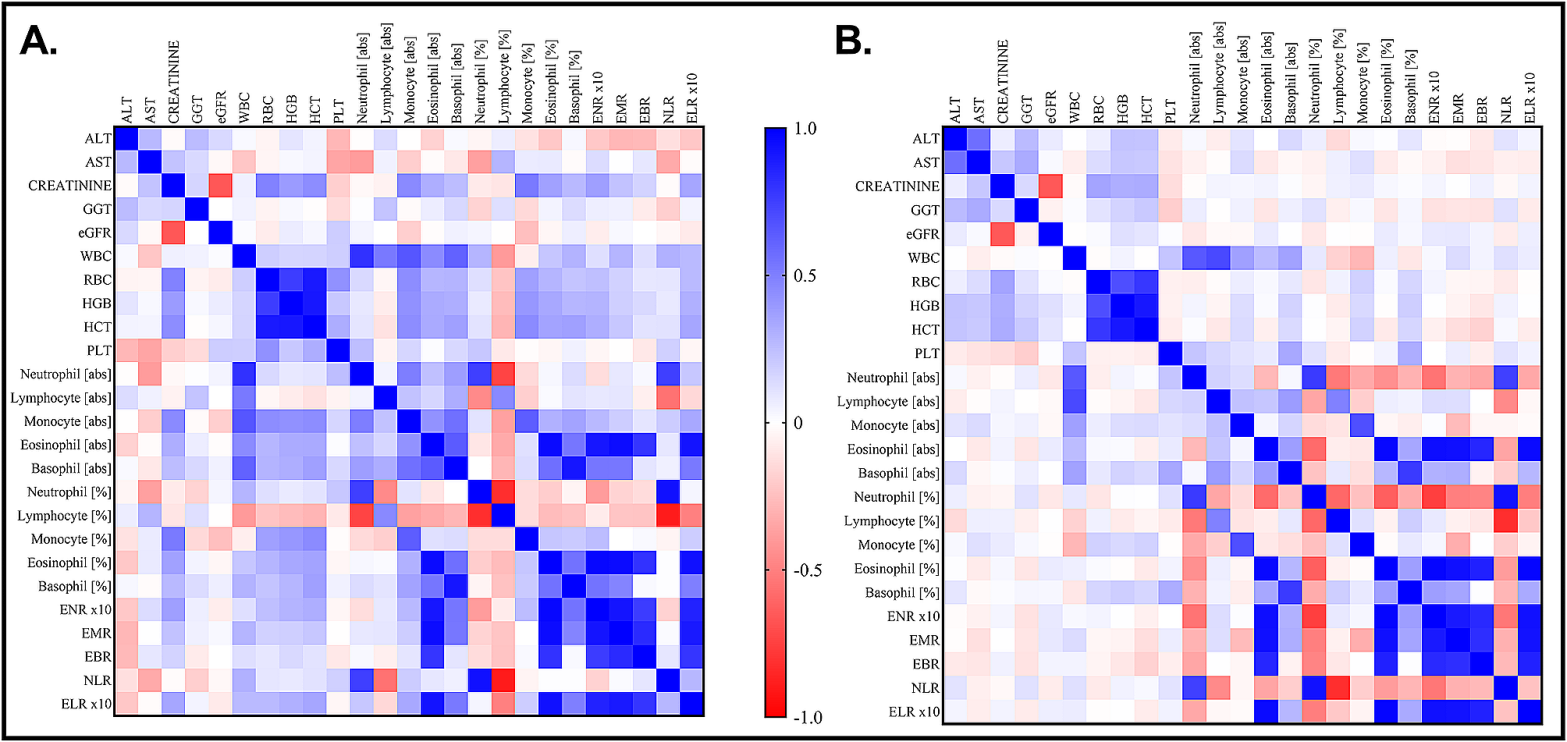
Spearman’s correlation analysis of BCRs with haemato-biochemical indices. (**A**) Correlation analysis among MF- participants (**B**) Correlation analysis among MF + participants. Blue-red coloration represents max (+) to min (-) correlation coefficient. RBC = red blood cell; HGB = haemoglobin concentration; HCT = haematocrit; PLT = platelet; WBC = white blood cell; ALT = alanine transaminase; AST = aspartate transaminase; GGT = gamma-glutamyl transferase; eGFR = estimated glomerular filtration; ENR: eosinophil-to-neutrophil ratio; EBR: eosinophil-to-basophil ratio; EMR: eosinophil-to-monocyte ratio; NLR: neutrophil-to-lymphocyte ratio; ELR: eosinophil-to-lymphocyte ratio (See details in Additional file 1 and Additional file 2)

Among the MF- participants, ENR x10, EMR, EBR and ELR x10 showed a significant positive correlation with absolute eosinophil and eosinophil percent (Fig. 4A). ENR x10, EMR, ELR x10 and not EBR showed a significant positive correlation with absolute basophil and basophil percent (Fig. 4A). ENR showed a significant negative correlation with neutrophil percent whiles ELR x10 showed a negative correlation with lymphocyte percent (Fig. 4A). ENR and ELR showed a significant positive correlation with creatinine (Fig. 4A). The numerical outcome of Spearman’s correlation analysis can be found in the supplementary sheet as supporting information.

Among the MF + participants, ENR x10, EMR, EBR and ELR x10 showed a significant positive correlation with absolute eosinophil and eosinophil percent but a negative correlation with absolute neutrophil and neutrophil percent (Fig. 4B). ENR x10, EMR and ELR x10 showed a significant positive correlation with absolute basophil and basophil percent (Fig. 4B). EMR showed a significant negative correlation with absolute monocyte and monocyte percent whiles ELR x10 showed a negative correlation with lymphocyte percent (Fig. 4B). Both absolute eosinophil and eosinophil percent showed a significant negative correlation with absolute neutrophil and neutrophil percent (Fig. 4B). Absolute eosinophil and eosinophil percent showed a significant positive correlation with absolute basophil and basophil percent (Fig. 4B). The numerical outcome of Spearman’s correlation analysis can be found in the supplementary sheet as supporting information.

### BCRs show fair predictability in classifying MF + and a-MF participants

**Fig. 5:**
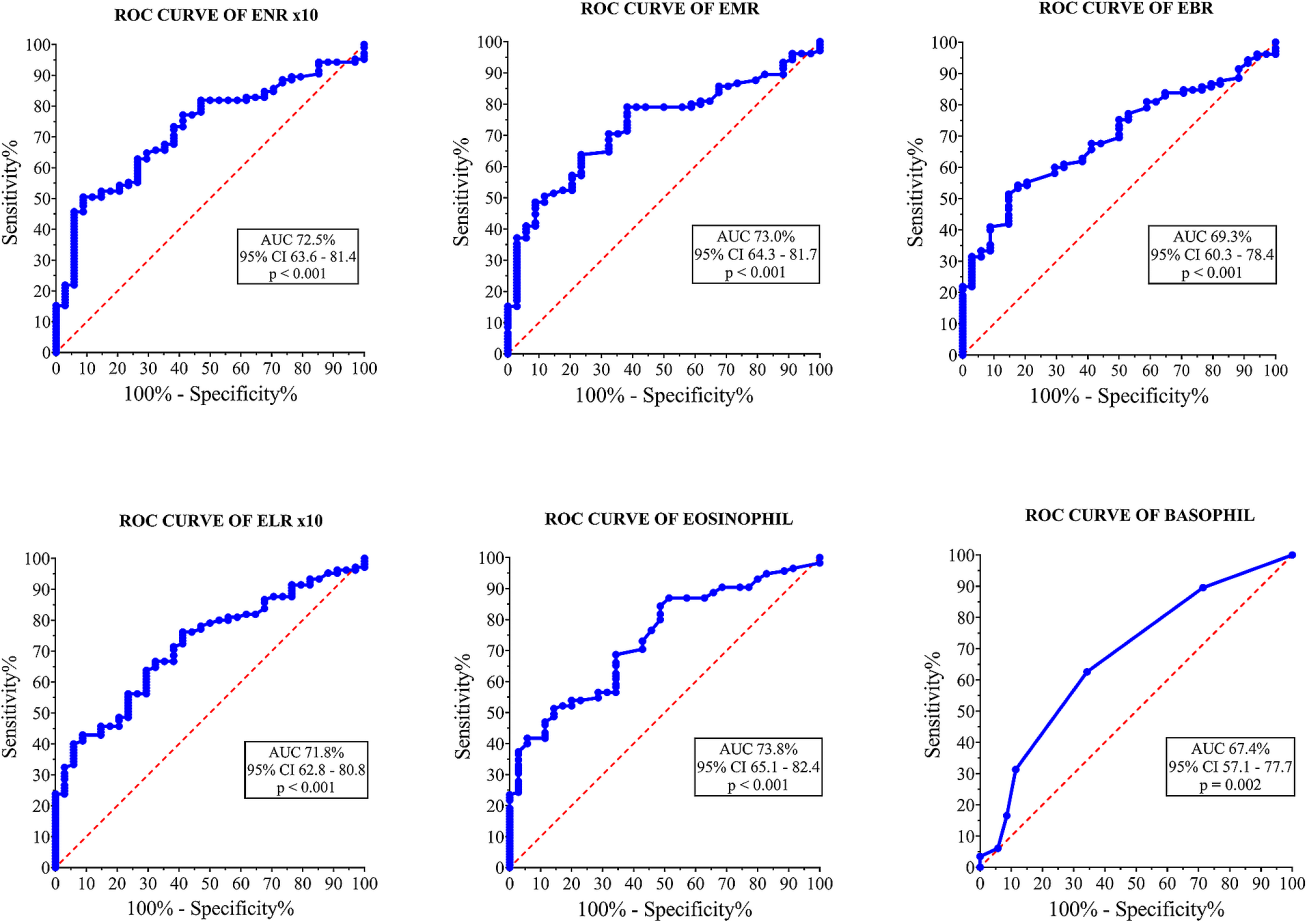
Predictability of blood composite ratios and inflammatory cells in *O. volvulus* infection ROC was based on binary logistic regression and discriminant classification analysis for MF + and MF- participants. AUC: area under the curve of ROC; CI: confidence interval

Receiver operator characteristic (ROC) curve analysis were performed to determine the diagnostic ability of BCRs in classifying MF + participants and MF- participants (Fig. 5). The analysis showed that ENR x10 (AUC = 72.5%; *p* < 0.001), EMR (AUC = 73.0%; *p* < 0.001) and ELR x10 (AUC = 71.8%; *p* < 0.001) had a fair predictive value in classifying MF + and MF- participants. Absolute eosinophil (AUC = 73.8%; *p* < 0.001) had the highest predictive values in discriminating onchocerciasis patients with microfilaridermia and a-microfilaridermia than either of the blood composite ratios (Fig. 5). EBR and basophil had the lowest predictive values of 69.3% and 67.4% respectively. For optimal cutoff values of BCRs that best distinguished between MF + and MF-, an ENR x10 > 2.265, EBR > 14.25, and absolute eosinophil > 0.415 showed higher specificity but lower sensitivity (Table 4). The optimal cutoff values of EMR that best distinguished between MF + and MF- were values > 0.395 with 79.1% sensitivity and 61.8% specificity (Table 4). ELR x10 cutoff value > 1.160 showed 63.8% sensitivity and 70.6% specificity in predicting onchocerciasis patients with microfilaridermia (Table 4).

**Table 4:** Cutoff values of blood composite ratios in predicting microfilaridermia

Parameter	Cut-off	Sensitivity (95% CI)	Specificity (95% CI)	Accuracy (95% CI)	PPV (95% CI)	NPV (95% CI)	*p*-Value
Eosinophil [Abs]	> 0.415	50.5 [40.6–60.4]	88.2 [72.6–96.7]	59.7 [51.1–67.9]	93.0 [83.8–97.1]	36.6 [31.5–42.0]	< 0.001
Basophil [Abs]	> 0.025	63.8 [53.9–73.0]	67.7 [49.5–82.6]	64.8 [56.2–72.7]	85.9 [78.6–91.0]	37.7 [30.0–46.1]	0.001
ENR x10	> 2.265	50.5 [40.6–60.4]	91.2 [76.3–98.1]	60.4 [51.8–68.6]	94.6 [85.5–98.2]	37.4 [32.4–42.6]	< 0.001
EMR	> 0.395	79.1 [70.0–86.4]	61.8 [43.6–77.8]	74.8 [66.8–81.8]	86.5 [80.5–90.8]	48.8 [37.7–60.1]	< 0.001
EBR	> 14.25	51.4 [41.5–61.3]	85.3 [68.9–95.1]	59.7 [51.1–67.9]	91.5 [82.5–96.1]	36.3 [30.9–42.0]	< 0.001
ELR x10	> 1.160	63.8 [53.9–73.0]	70.6 [52.5–84.9]	65.5 [56.9–73.3]	87.0 [79.6–92.0]	38.5 [31.1–46.9]	< 0.001

PPV; positive predictive value, NPV; negative predictive value, CI; confidence interval, abs; absolute count, EBR: eosinophil-to-basophil ratio; EMR: eosinophil-to-monocyte ratio; NLR: neutrophil-to-lymphocyte ratio; ELR: eosinophil-to-lymphocyte ratio. The Chi-square test was used for all analyses. *p*-value less than 0.05 was considered statistically significant

### Eosinophil count and BCRs are independently associated with increased odds of microfilaridermia in Onchocerciasis

Univariate analysis showed that ENR x10 (cOR = 1.60, 95% CI [1.18–2.19, *p* = 0.003), EMR (cOR = 3.68, 95% CI [1.64–8.26], *p* = 0.002), EBR (cOR = 1.09, 95% CI [1.03–1.15], *p* = 0.003) and ELR x10 (cOR = 1.93, 95% CI [1.29–2.88], *p* = 0.001) were significantly associated with increased odds of microfilariae positivity (Table 5). After adjusting for age, gender, years lived in endemic area, distance to the nearest river, occupation, individual IVM intake, total nodule counts, total nodule sites, ENR x10 (aOR = 1.42, 95% CI [1.05–1.93], *p* = 0.025), EMR (aOR = 2.64, 95% CI [1.25–5.60], *p* = 0.011), EBR (aOR = 1.07, 95% CI [1.01–1.10], *p* = 0.020) and ELR x10 (aOR = 1.69, 95% CI [1.14–2.51], *p* = 0.009) were independently associated with increased odds of microfilariae positivity (Table 6). High eosinophil counts were independently associated with microfilariae positivity (aOR = 13.86, 95% CI [2.07–92.90], *p* = 0.007) (Table 6).

**Table 5:** Univariate logistics regression analysis for microfilaridermia among study participants

Variable	Univariate analysis	
	OR (95 CI %)	*p*-value
Age (years)	0.97 [0.93–1.01]	0.121
Year in an endemic area (years)	0.96 [0.92–0.99]	**0.011**
gender (male)	1.09 [0.48–2.46]	0.834
Distance to the river (< 1 km)	0.33 [0.07–1.59]	0.165
Distance to the river (1–2 km)	0.18 [0.04–0.85]	**0.030**
Occupation (Farmer)	2.66 [1.09–6.51]	**0.032**
Individual IVM intake	0.79 [0.61–1.02]	0.080
Total nodule sites	1.54 [0.89–2.66]	0.124
Total nodule counts	1.11 [0.85–1.45]	0.425
Total WBC	1.74 [1.20–2.51]	**0.003**
Basophil	1.65 [1.16–2.34]	**0.005**
Eosinophil	28.18 [3.97–200.13]	**0.001**
GGT	0.98 [0.96–1.00]	0.067
eGFR	1.02 [1.00–1.05]	0.062
ENR x10	1.60 [1.18–2.19]	**0.003**
EMR	3.68 [1.64–8.26]	**0.002**
EBR	1.09 [1.03–1.15]	**0.003**
ELR x10	1.93 [1.29–2.88]	**0.001**

WBC; white blood cell count, GGT; gamma glutamyl transferase; ENR; eosinophil-to-neutrophil ratio, EMR; eosinophil-to-monocyte ratio, EBR; eosinophil-to-basophil ratio, ELR; eosinophil-to-lymphocyte ratio

**Table 6:** Multivariate logistics regression analysis for microfilaridermia among study participants

Variable	Multivariate analysis					
	Model 1 + ENR		Model 1 + EMR		Model 1 + EBR	
	aOR (95 CI %)	*p*-value	aOR (95 CI %)	*p*-value	aOR (95 CI %)	*p*-value
Age (years)	0.99 [0.93–1.05]	0.687	0.99 [0.93–1.05]	0.676	0.99 [0.93–1.05]	0.688
gender (male)	0.61 [0.20–1.80]	0.367	0.65 [0.22–1.92]	0.438	0.60 [0.20–1.80]	0.361
Year in an endemic area (years)	0.97 [0.92–1.01]	0.162	0.97 [0.92–1.02]	0.174	0.96 [0.92–1.01]	0.105
Distance to the river (< 1 km)	0.29 [0.05–1.68]	0.167	0.26 [0.04–1.50]	0.130	0.31 [0.05–1.76]	0.185
Distance to the river (1–2 km)	0.18 [0.03–1.02]	0.052	0.15 [0.03–0.84]	**0.031**	0.17 [0.03–0.97]	**0.046**
Occupation (Farmer)	5.67 [1.58–20.33]	**0.008**	4.97 [1.41–17.56]	**0.013**	6.01 [1.67–21.59]	**0.006**
Individual IVM intake	0.90 [0.65–1.25]	0.526	0.92 [0.51–1.49]	0.563	0.92 [0.66–1.28]	0.619
Total nodule counts	0.94 [0.58–1.52]	0.791	0.92 [0.57–1.49]	0.745	0.91 [0.56–1.46]	0.687
Total nodule sites	1.52 [0.57–4.08]	0.408	1.48 [0.55–4.02]	0.441	1.48 [0.55–3.99]	0.436
ENR x10	1.42 [1.05–1.93]	**0.025**				
EBR			2.64 [1.25–5.60]	**0.011**		
EMR x10					1.07 [1.01–1.10]	**0.020**
	**Multivariate analysis**					
	**Model 1 + ELR**		**Model 1 + Eosinophil**			
	**aOR (95 CI %)**	***p***-**value**	**aOR (95 CI %)**	***p***-**value**		
Age (years)	0.99 [0.93–1.06]	0.654	0.99 [0.93–1.05]	0.696		
gender (male)	0.56 [0.19–1.69]	0.304	0.55 [0.18–1.71]	0.300		
Year in an endemic area (years)	0.97 [0.92–1.02]	0.177	0.97 [0.92–1.02]	0.196		
Distance to the river (< 1 km)	0.28 [0.05–1.66]	0.163	0.25 [0.04–1.50]	0.126		
Distance to the river (1–2 km)	0.16 [0.03–0.91]	0.039	0.16 [0.03–0.93]	0.041		
Occupation (Farmer)	5.94 [1.65–21.39]	**0.006**	5.74 [1.55–21.25]	0.009		
Individual IVM intake	0.91 [0.65–1.27]	0.566	0.92 [0.66–1.30]	0.646		
Total nodule counts	0.93 [0.57–1.49]	0.751	0.91 [0.56–1.49]	0.709		
Total nodule sites	1.43 [0.53–3.87]	0.484	1.52 [0.56–4.15]	0.411		
ELR x10	1.69 [1.14–2.51]	**0.009**				
Eosinophil [abs]			13.86 [2.07–92.90]	**0.007**		

Model 1 included age, gender, years lived in an endemic area, distance to the nearest river, occupation, total nodule counts, total nodule sites, ENR; eosinophil-to-neutrophil ratio, EMR; eosinophil-to-monocyte ratio, EBR; eosinophil-to-basophil ratio, ELR; eosinophil-to-lymphocyte ratio

### ENR, EMR, EBR and ELR significantly improve the conventional model for predicting microfilaridermia

To verify whether the addition of BCRs to a model containing conventional predictors could improve the classification of MF + and MF- onchocercomata participants, C-statistics were used (Table 7). The results show that eosinophil, ENR, EMR, EBR and ELR significantly improved the model by 4.7%, 4.2%, 4.3%, 3.3% and 3.6% respectively (Table 7). Combining all the BCRs significantly led to the improvement of the model by 4.0% (AUC = 81.8%, 95% CI (74.2–89.4), *p* < 0.0001) for predicting microfilaridermic participants. Strikingly, adding eosinophil to the combined BCRs showed the best discriminating ability of the model with an AUC of 82.9% (95% CI, 75.6–90.2, *p* < 0.0001).

**Table 7:** C-statistics for blood ratios to improve the predictability of microfilaridermia among study participants

Parameter	C-statistics		
	Estimate	95% CI	*p*-value
Model 1	0.778	0.692–0.864	< 0.0001
Model 1 + Eosinophil	0.825	0.752–0.899	< 0.0001
Model 1 + ENR	0.820	0.740–0.899	< 0.0001
Model 1 + EMR	0.821	0.745–0.897	< 0.0001
Model 1 + EBR	0.811	0.733–0.889	< 0.0001
Model 1 + ELR	0.814	0.739–0.889	< 0.0001
Model 1 + ENR + EMR + EBR + ELR	0.818	0.742–0.894	< 0.0001
Model 1 + Eosinophil + Combined ratios	0.829	0.756–0.902	< 0.0001

Model 1 was defined to include age, gender (male), years lived in an endemic area, distance to the nearest river, occupation (farmer), individual intake of MDA (ivermectin), total nodule counts and total nodule sites. ENR; eosinophil-to-neutrophil ratio, EMR; eosinophil-to-monocyte ratio, EBR; eosinophil-to-basophil ratio, ELR; eosinophil-to-lymphocyte ratio; combined ratios included ENR, EMR, EBR and ELR

## Discussion

Attempts are being made to distinguish between onchocerciasis subgroups that have unique prognostic characteristics or display distinct responses to treatment due to the ongoing effects of CDTI [13, 16, 20]. Onchocerciasis causes chronic systemic inflammation even in patients with few pathologies. Various diagnostic markers have been used to detect systemic inflammation in patients including haemato-biochemical indices [30, 31]. Previous studies have shown that total WBC, neutrophil, eosinophil, basophil and monocytes play an important role and are used as indicators of an inflammatory response [30, 32]. In our study, we observed significantly higher total WBC, eosinophil and basophil counts in MF + participants implying an ongoing inflammatory reaction. Again, we observed that MF + participants had significantly higher eosinophils but less neutrophil counts compared to MF- participants corroborating previous findings [13, 20].

The enzyme gamma-glutamyl transferase (GGT) is mostly present in the liver and is important for maintaining intracellular glutathione levels [33, 34]. It is frequently employed as a marker for liver or biliary disorders [33]. Elevated GGT is linked to an increased risk of many chronic diseases, including cardiovascular disease, diabetes, metabolic syndrome and chronic infection [35–37]. However, our study found that reduced GGT levels were significantly associated with MF + participants compared to MF- participants. The mechanism underlying this is unknown, and further research is needed to better understand the function of GGT in onchocerciasis subgroups and how it affects the inflammatory immune response.

Evaluation of glomerular filtration rate (GFR) is critical for the diagnosis and treatment of kidney diseases [38, 39]. The estimated GFR (eGFR), one of the several ways to measure GFR, is recommended for the initial evaluation of GFR [40]. Observational studies have shown that declining eGFR values are associated with an increased risk of kidney complications, cardiovascular diseases and mortality [39, 41]. Several parasitic infections have been associated with glomerular disease [42–44]. Direct parasite destruction, immunological consequences, and systemic symptoms all play a role in the mechanism underlying parasite-induced kidney disease [45]. A study showed that onchocerciasis patients have a high prevalence of glomerular and tubular disturbances [46]. In this study, we observed a significantly lower eGFR among MF- participants compared to MF + participants. This could be due to immune complex-mediated damage caused by dead parasites among MF- participants [44, 45, 47].

Studies show that occupation, distance to the nearest river, age, compliance to the MDA programme, presence of nodules and gender are conventional predictors of onchocerciasis infections [13, 28, 48, 49]. We observed that farming was associated with an increased likelihood of microfilaridermia in our study before and after adjusting for covariables. Infectious bites from vectors may occur repeatedly during active farming periods, which typically are reported in endemic areas [50]. Kifle et al. showed that participants who lived < 2 km from the nearest river were more likely to have microfilaridermia compared to those who lived ≥ 2 km from the nearest river [51]. Our study observed that individuals who lived 1–2 km from the nearest river were less likely to have microfilaridermia compared to those who lived > 2 km away from the river. This could be due to the observed significantly higher intake of IVM among participants who lived nearer to the river than those far away in our study. Treatment coverage and compliance are highly effective at reducing the microfilariae burden at both community and individual levels [1, 52].

Centering on onchocerciasis infection, Tamarozzi et al., reported that inflammatory reaction to dead skin MF and MF in the eyes alongside the subsequent release of their bacterial endosymbiont *Wolbachia* was the basis of *Onchocerca* dermatitis and ocular keratitis immunopathology [18]. Microfilariae from *Onchocerca* dermatitis tissues have been reported to be surrounded by neutrophils, eosinophils or macrophages [53, 54]. Previous studies have emphasized the association between eosinophilia and the severity of onchocerciasis [55–57]. Eosinophil infiltration into onchocercomata is triggered by MF and it has been shown that eosinophils actively attack MF [58, 59]. Evidence shows that live adult *O. volvulus* does not elicit eosinophilic invasion so long as they do not produce MF [58]. In chronically infected and untreated participants, eosinophil and macrophages infiltration cause permanent tissue damage [18]. Our study corroborates these hypotheses where we observed disproportionate abnormalities in eosinophil levels (51.4%) which were significantly associated with increased odds of microfilaridermia in MF + participants.

Focusing on basophil involvement in onchocerciasis infection, our findings corroborate the evidence that basophil numbers are increased in helminth infection [60, 61]. Basophils have been shown to release histamine and interleukin 4 (IL-4) in response to filarial nematodes where they function to amplify the ongoing type 2 immune response [62]. Furthermore, it has been shown that depletion of basophils results in a drastic reduction of eosinophils proliferation [62]. Labadah et al. reported a significantly lower basophil count with high eosinophil count in MF + participants compared to MF- participants [20]. In our study, MF + participants had significantly higher basophil compared to MF- participants. This may be because their participants were relatively younger than those observed in our study and a study showed that age significantly influences changes in neutrophil, basophil and eosinophil immunity [63].

Neutrophils are the major effector cells of early inflammatory infiltrate around damaged MF in the cornea and skin or attached to *Wolbachia*-containing adult worms [64, 65]. Their recruitment, activation and subsequent development of tissue pathology or systemic adverse events to a microfilaricidal drug depend on the endosymbiont *Wolbachia* [18]. Brattig et al. found that following doxycycline depletion of *Wolbachia*, the neutrophil infiltrates in onchocercomata were drastically reduced [64]. Although their role remains debatable, recent studies suggest that they may be involved in host protective immunity through *Wolbachia*-induced neutrophil extracellular trap formation (NET) [65, 66]. The function of this NETosis limits the dissemination and prevention of inflammatory damage induced by *Wolbachia*; provides an anti-parasitic response to limit MF production; and also limits the penetration of more damaging immune cells such as degranulating eosinophils [66]. Previously, we reported significantly higher levels of neutrophils among MF- participants compared to MF + participants [13]. A similar trend was observed in this study but was not statistically significant. This could be because of the homeostatic restoration of neutrophil activation after a limited period of IVM treatment [67, 68]. Neutrophil activation is observed during the adverse reaction following treatment with IVM and correlates with MF load, presence of *Wolbachia* DNA and proinflammatory cytokines [69]. Levels of neutrophils increase shortly after IVM and are reduced to baseline after 4–6 months of *Wolbachia* depletion [68–70].

Different from previous studies, we evaluated the blood composite ratios (BCRs) of MF + and MF- participants based on reported interaction between eosinophil and other inflammatory cells in discriminating onchocerciasis subgroups [13, 20]. We observed significantly higher levels of ENR, EBR, EMR and ELR in the MF + participants compared to MF- participants. We also found that elevated ENR, EMR, EBR and ELR correlated positively with eosinophil but negatively with neutrophil in MF + participants. The clinical implications of these composite ratios have been reported particularly in inflammatory diseases, allergies and cancers. Higher ELR values were associated with worse survival results, according to Holub and Biete [26], who reported an AUROC of 0.61 for utilizing ELR to predict survival outcomes. Brescia et al., also observed that EBR was significantly higher in patients with inflammatory diseases [71]. These ratios have proved to be better prediction biomarkers than established biomarkers [72]. The increased ENR, EBR, EMR and ELR in this study suggest that they might serve as stable complementary biomarkers to eosinophils in predicting microfilariae in *O. volvulus* infection. ENR > 0.2265, EBR > 14.25 and ELR > 0.116 showed significantly higher specificity (> 80%) in diagnosing microfilaridermias. This is important to the current elimination goals of onchocerciasis, where ensuring a very high degree of specificity is paramount [73]. EMR is a stable biomarker [74] and proved to be very robust in discriminating between MF + and MF- participants in this study. At a level of > 0.395, EMR showed considerably higher specificity and sensitivity in detecting skin microfilariae. The reported specificity and sensitivity of these biomarkers corroborate reports on sensitivity and specificity by other researchers who explored these biomarkers [26, 74, 75]. Interestingly, we found that eosinophil alone had the highest AUROC value (73.8%) and presented much better accuracy than all the BCRs. The C-statistics analysis revealed that these markers should be considered as part of the conventional model of diagnosing microfilaridermia among onchocercomata patients and not as a standalone biomarker. In endemic areas with variable levels of medical resources, a routine complete blood count test is offered as part of the diagnosis. BCRs could be obtained from a simple calculation of absolute cell counts and may assist clinicians to judge whether patients will harbour microfilariae.

Our study is not without limitations. Even though according to the Ghana NTD Control Programme and other studies there may not be other filarial infections in the district [76–78], we cannot exclusively reject the possibility of Onchocerciasis co-infection with other filarial diseases such LF and Mansonellosis in the participants recruited, but the chances are very low considering the site (Sefwi Akontombra district) where this study was carried out, which is the western North Region of Ghana. Also, as a retrospective study, it is burdened by all the associated limitations that accompany this type of data sampling method including an absence of data on potential confounders. Although the observed changes in blood ratios do not solely indicate microfilarial presence, they reflect the intricate interplay between eosinophils and the immune response. Therefore, their interpretation should be context specific. Notwithstanding, the results of our study should be considered as hypothesis generating as it has not been previously elucidated in this study population.

We do recommend that further confirmatory study in a sufficiently powered prospective analysis be done which considers the above-mentioned limitations.

## Conclusion

To conclude, we observed a significantly higher total WBC, absolute eosinophil, absolute basophil, ENR, EBR, EMR and ELR levels in the MF + participants compared to MF- participants. We also found that ENR, EBR, EMR and ELR were significantly associated with increased odds of MF positivity in participants with onchocercomata. Combining BCRs with eosinophil significantly led to improvement in the conventional model for predicting microfilaridermia.

### Electronic supplementary material

Below is the link to the electronic supplementary material.

Supplementary Material 1

Supplementary Material 2

## Data Availability

The datasets used and/or analysed during the current study are available from the corresponding author on reasonable request.

## Abbreviations

MF+: Microfilaria positive
MF-: Microfilaria negative
BCRs: Blood composite ratios
MDA: Mass Drug Administration
GGT: Gamma-glutamyl transferase
eGFR: estimated glomerular filtration rate
ENR: Eosinophil-to-neutrophil ratio
EMR: Eosinophil-to-monocyte ratio
EBR: Eosinophil-to-basophil ratio
ELF: Eosinophil-to-lymphocyte ratio
CDTI: Community-directed treatment with ivermectin
WHO: World Health Organization
IVM: Ivermectin
ALT: Alanine transaminase
AST: Aspartate transaminase
NPV: Negative predictive values
PPV: Positive predictive values

## References

[1] Turner HC, Walker M, Churcher TS, Basáñez MG. Modelling the impact of ivermectin on River Blindness and its burden of morbidity and mortality in African Savannah: EpiOncho projections. Parasites and Vectors [Internet]. 2014 May 26 [cited 2021 Nov 11];7(1):1–15. https://link.springer.com/articles/10.1186/1756-3305-7-241. 10.1186/1756-3305-7-241 PMC4037555 24886747

[2] Vinkeles Melchers NVS, Stolk WA, van Loon W, Pedrique B, Bakker R, Murdoch ME et al. The burden of skin disease and eye disease due to onchocerciasis in countries formerly under the african programme for onchocerciasis control mandate for 1990, 2020, and 2030. PLoS Negl Trop Dis [Internet]. 2021 Jul 1 [cited 2021 Nov 11];15(7):e0009604. https://journals.plos.org/plosntds/article?id=10.1371/journal.pntd.0009604. 10.1371/journal.pntd.0009604 PMC8312930 34310602

[3] WHO. Onchocerciasis [Internet]. 2019 [cited 2021 Nov 11]. https://www.who.int/news-room/fact-sheets/detail/onchocerciasis.

[4] Okulicz JF, Elston DM, Schwartz RA. Dermatologic Manifestations of Onchocerciasis (River Blindness): Background, Pathophysiology, Etiology [Internet]. https://emedicine.medscape.com/article/1109409-overview#section~clinical. 2018 [cited 2021 Nov 11]. https://emedicine.medscape.com/article/1109409-overview#section~clinical.

[5] World Health Organization. Ending the neglect to attain the Sustainable Development Goals: a road map for neglected tropical diseases 2021–2030. Geneva: World Health Organization (https://www.who.int/neglected_diseases/Revised-DraftNTD-Roadmap-23Apr2020.pdf) [Internet]. 2020 [cited 2021 Nov 11]. 196 p. https://apps.who.int/iris/bitstream/handle/10665/332094/WHO-UCN-NTD-2020.01-eng.pdf.

[6] Gebrezgabiher G, Mekonnen Z, Yewhalaw D, Hailu A. Status of parasitological indicators and morbidity burden of onchocerciasis after years of successive implementation of mass distribution of ivermectin in selected communities of Yeki and Asosa districts, Ethiopia. BMC Public Health [Internet]. 2020 Aug 12 [cited 2021 Nov 11];20(1):1–15. https://link.springer.com/articles/10.1186/s12889-020-09344-7. 10.1186/s12889-020-09344-7 PMC7425055 32787813

[7] Katabarwa MN, Habomugisha P, Eyamba A, Byamukama E, Nwane P, Arinaitwe A et al. Community-directed interventions are practical and effective in low-resource communities: Experience of ivermectin treatment for onchocerciasis control in Cameroon and Uganda, 2004–2010. Int Health [Internet]. 2015 Mar 1 [cited 2021 Nov 11];8(2):116–23. https://academic.oup.com/inthealth/article/8/2/116/2458791. 10.1093/inthealth/ihv038 26152231

[8] Forrer A, Wanji S, Obie ED, Nji TM, Hamill L, Ozano K et al. Why onchocerciasis transmission persists after 15 annual ivermectin mass drug administrations in South-West Cameroon. BMJ Glob Heal [Internet]. 2021 [cited 2021 Nov 11];6(1). https://gh.bmj.com/content/6/1/e003248.abstract. 10.1136/bmjgh-2020-003248 PMC7802695 33431378

[9] Colebunders R, Stolk WA, Siewe Fodjo JN, Mackenzie CD, Hopkins A. Elimination of onchocerciasis in Africa by 2025: An ambitious target requires ambitious interventions [Internet]. Vol. 8, Infectious Diseases of Poverty. BioMed Central Ltd.; 2019 [cited 2021 Nov 11]. pp. 1–3. https://idpjournal.biomedcentral.com/articles/10.1186/s40249-019-0593-x. 10.1186/s40249-019-0593-x PMC6775645 31578157

[10] Brattig NW. Pathogenesis and host responses in human onchocerciasis: impact of Onchocerca filariae and Wolbachia endobacteria. Microbes and Infection. Volume 6. Elsevier Masson; 2004. pp. 113–28. 10.1016/j.micinf.2003.11.003 14738900

[11] Soboslay PT, Luder CGK, Hoffmann WH, Michaelis I, Helling G, Heuschkel C et al. Ivermectin-facilitated immunity in onchocerciasis: Activation of parasite- specific Th1-type responses with subclinical Onchocerca volvulus infection. Clin Exp Immunol [Internet]. 1994 [cited 2021 Nov 11];96(2):238–44. https://pubmed.ncbi.nlm.nih.gov/8187332/. 10.1111/j.1365-2249.1994.tb06548.x PMC1534906 8187332

[12] Lechner CJ, Gantin RG, Seeger T, Sarnecka A, Portillo J, Schulz-Key H et al. Chemokines and cytokines in patients with an occult Onchocerca volvulus infection. Microbes Infect [Internet]. 2012 [cited 2021 Nov 11];14(5):438–46. https://www.sciencedirect.com/science/article/pii/S1286457911003066. 10.1016/j.micinf.2011.12.002 22202179

[13] Arndts K, Specht S, Debrah AY, Tamarozzi F, Klarmann Schulz U, Mand S et al. Immunoepidemiological profiling of Onchocerciasis patients reveals associations with Microfilaria loads and Ivermectin Intake on both individual and community levels. PLoS Negl Trop Dis. 2014;8(2). 10.1371/journal.pntd.0002679 PMC3930501 24587458

[14] Makepeace BL, Martin C, Turner JD, Specht S. Granulocytes in Helminth Infection - who is calling the shots? Curr Med Chem [Internet]. 2012 Mar 9 [cited 2021 Nov 12];19(10):1567–86. Available from: /pmc/articles/PMC3394172/. 10.2174/092986712799828337 PMC3394172 22360486

[15] Taylor MJ, Hoerauf A, Bockarie M. Lymphatic filariasis and onchocerciasis. In: The Lancet [Internet]. Elsevier; 2010 [cited 2021 Nov 11]. pp. 1175–85. http://www.thelancet.com/article/S0140673610605867/fulltext. 10.1016/S0140-6736(10)60586-7 20739055

[16] Katawa G, Layland LE, Debrah AY, von Horn C, Batsa L, Kwarteng A et al. Hyperreactive Onchocerciasis is Characterized by a Combination of Th17-Th2 Immune Responses and Reduced Regulatory T Cells. PLoS Negl Trop Dis [Internet]. 2015 [cited 2021 Nov 12];9(1):e3414. https://journals.plos.org/plosntds/article?id=10.1371/journal.pntd.0003414. 10.1371/journal.pntd.0003414 PMC4288720 25569210

[17] Voehringer D. The role of basophils in helminth infection. Trends in Parasitology. Volume 25. Elsevier Current Trends; 2009. pp. 551–6. 10.1016/j.pt.2009.09.004 19782643

[18] Tamarozzi F, Halliday A, Gentil K, Hoerauf A, Pearlman E, Taylor MJ. Onchocerciasis: The role of Wolbachia bacterial endosymbionts in parasite biology, disease pathogenesis, and treatment. Clin Microbiol Rev [Internet]. 2011 Jul [cited 2021 Nov 12];24(3):459–68. 10.1128/CMR.00057-10. 10.1128/CMR.00057-10 PMC3131055 21734243

[19] Arndts K, Klarmann-Schulz U, Batsa L, Debrah AY, Epp C, Fimmers R et al. Reductions in microfilaridermia by repeated ivermectin treatment are associated with lower Plasmodium-specific Th17 immune responses in Onchocerca volvulus-infected individuals. Parasites and Vectors [Internet]. 2015 Mar 28 [cited 2021 Nov 12];8(1):1–10. https://parasitesandvectors.biomedcentral.com/articles/10.1186/s13071-015-0786-5. 10.1186/s13071-015-0786-5 PMC4391604 25889652

[20] Labadah J, Kusi KA, Wilson M. Comparison of some immunological parameters between untreated and ivermectin-treated onchocerciasis patients in the Nkwanta North District of Ghana. Int J Infect Dis [Internet]. 2020 Dec 1 [cited 2022 Jan 5];101(S1):433. http://www.ijidonline.com/article/S1201971220318518/fulltext.

[21] Ramos-Lopez O, San-Cristobal R, Martinez-Urbistondo D, Micó V, Colmenarejo G, Villares-Fernandez P et al. Proinflammatory and hepatic features related to morbidity and fatal outcomes in covid-19 patients. J Clin Med [Internet]. 2021 Jul 15 [cited 2021 Nov 12];10(14):3112. https://www.mdpi.com/2077-0383/10/14/3112/htm. 10.3390/jcm10143112 PMC8306049 34300279

[22] Ciccullo A, Borghetti A, Zileri Dal Verme L, Tosoni A, Lombardi F, Garcovich M et al. Neutrophil-to-lymphocyte ratio and clinical outcome in COVID-19: a report from the Italian front line. Int J Antimicrob Agents [Internet]. 2020 Aug 1 [cited 2021 Nov 12];56(2):106017. /pmc/articles/PMC7211594/. 10.1016/j.ijantimicag.2020.106017 PMC7211594 32437920

[23] Karauda T, Kornicki K, Jarri A, Antczak A, Miłkowska-Dymanowska J, Piotrowski WJ et al. Eosinopenia and neutrophil-to-lymphocyte count ratio as prognostic factors in exacerbation of COPD. Sci Reports 2021 111 [Internet]. 2021 Feb 26 [cited 2021 Nov 12];11(1):1–9. https://www.nature.com/articles/s41598-021-84439-8. 10.1038/s41598-021-84439-8 PMC7910289 33637803

[24] Cai H, Huang H, Yang C, Ren J, Wang J, Gao B et al. Eosinophil-to-neutrophil ratio predicts poor prognosis of Acute ischemic stroke patients treated with intravenous thrombolysis. Front Neurol. 2021;12. 10.3389/fneur.2021.665827 PMC8310951 34322078

[25] Hota PK, Reddy BG. Role of eosinophil count and neutrophil: lymphocyte count ratio as prognostic markers in patients with sepsis. Int Surg J [Internet]. 2017 Jun 22 [cited 2021 Nov 12];4(7):2243. https://ijsurgery.com/index.php/isj/article/view/1324.

[26] Holub K, Biete A. New pre-treatment eosinophil-related ratios as prognostic biomarkers for survival outcomes in endometrial cancer. BMC Cancer [Internet]. 2018 Dec 22 [cited 2021 Nov 12];18(1):1–9. https://bmccancer.biomedcentral.com/articles/10.1186/s12885-018-5131-x. 10.1186/s12885-018-5131-x PMC6304088 30577833

[27] Ghana Statistical Service (GSS). Ghana 2021 Population and Housing Census: General Analytical Report - Sefwi Akontombra. Available: www.statsghana.gov.gh.

[28] Duerr HP, Raddatz G, Eichner M. Diagnostic value of nodule palpation in onchocerciasis. Trans R Soc Trop Med Hyg [Internet]. 2008 Feb 1 [cited 2022 Jan 12];102(2):148–54. https://academic.oup.com/trstmh/article/102/2/148/1920767. 10.1016/j.trstmh.2007.10.009 18082234

[29] Levey AS, Coresh J, Greene T, Marsh J, Stevens LA, Kusek JW et al. Expressing the modification of diet in renal disease study equation for estimating glomerular filtration rate with standardized serum creatinine values. Clin Chem [Internet]. 2007 Apr [cited 2022 Jun 22];53(4):766–72. https://pubmed.ncbi.nlm.nih.gov/17332152/. 10.1373/clinchem.2006.077180 17332152

[30] Khalid A, Ali Jaffar M, Khan T, Abbas Lail R, Ali S, Aktas G et al. Hematological and biochemical parameters as diagnostic and prognostic markers in SARS-COV-2 infected patients of Pakistan: a retrospective comparative analysis. Hematol (United Kingdom) [Internet]. 2021 [cited 2022 Jun 22];26(1):529–42. https://www.tandfonline.com/doi/abs/10.1080/16078454.2021.1950898. 10.1080/16078454.2021.1950898 34334100

[31] Ndzeshang BL, Mbiakop RT, Nchanji GT, Kien CA, Amambo GN, Abong RA et al. Clinical, haematological and biochemical profiling of podoconiosis lymphoedema patients prior to their involvement in a clinical trial in the Northwest Region of Cameroon. Trans R Soc Trop Med Hyg [Internet]. 2020 Dec 16 [cited 2022 Jun 22];114(12):954–61. https://academic.oup.com/trstmh/article/114/12/954/6015483. 10.1093/trstmh/traa146 PMC7738657 33258944

[32] Aninagyei E, Nanga S, Acheampong DO, Mensah R, Boadu MN, Kwansa-Bentum HT et al. Prevalence and risk factors of human Balantidium coli infection and its association with haematological and biochemical parameters in Ga West Municipality, Ghana. BMC Infect Dis [Internet]. 2021 Dec 1 [cited 2022 Jun 22];21(1):1–10. https://link.springer.com/articles/10.1186/s12879-021-06731-2. 10.1186/s12879-021-06731-2 PMC8502288 34627168

[33] Koenig G, Seneff S, Gamma-Glutamyltransferase. A Predictive Biomarker of Cellular Antioxidant Inadequacy and Disease Risk. Vol. 2015, Disease Markers. Hindawi Limited; 2015. 10.1155/2015/818570 PMC4620378 26543300

[34] Kunutsor SK. Gamma-glutamyltransferase—friend or foe within? [Internet]. Vol. 36, Liver International. Liver Int; 2016 [cited 2022 Jun 22]. pp. 1723–34. https://pubmed.ncbi.nlm.nih.gov/27512925/. 10.1111/liv.13221 27512925

[35] Ndrepepa G, Kastrati A. Gamma-glutamyl transferase and cardiovascular disease. Ann Transl Med [Internet]. 2016 Dec 1 [cited 2022 Jun 22];4(24). /pmc/articles/PMC5233492/. 10.21037/atm.2016.12.27 PMC5233492 28149843

[36] Bo S, Gambino R, Durazzo M, Guidi S, Tiozzo E, Ghione F et al. Associations between gamma-glutamyl transferase, metabolic abnormalities and inflammation in healthy subjects from a population-based cohort: a possible implication for oxidative stress. World J Gastroenterol [Internet]. 2005 Dec 7 [cited 2022 Jun 22];11(45):7109–17. https://pubmed.ncbi.nlm.nih.gov/16437656/. 10.3748/wjg.v11.i45.7109 PMC4725082 16437656

[37] Silva ISS, Ferraz MLCG, Perez RM, Lanzoni VP, Figueiredo VM, Silva AEB. Role of γ-glutamyl transferase activity in patients with chronic hepatitis C virus infection. J Gastroenterol Hepatol [Internet]. 2004 Mar 1 [cited 2022 Jun 22];19(3):314–8. https://onlinelibrary.wiley.com/doi/full/10.1111/j.1440-1746.2003.03256.x. 10.1111/j.1440-1746.2003.03256.x 14748879

[38] Matsushita K, Selvin E, Bash LD, Astor BC, Coresh J (2010). Risk implications of the New CKD Epidemiology Collaboration (CKD-EPI) equation compared with the MDRD Study equation for estimated GFR: the atherosclerosis risk in communities (ARIC) Study. Am J Kidney Dis.

[39] Nitsch D, Grams M, Sang Y, Black C, Cirillo M, Djurdjev O et al. Associations of estimated glomerular filtration rate and albuminuria with mortality and renal failure by sex: A meta-analysis. BMJ [Internet]. 2013 Jan 29 [cited 2022 Jul 15];346(7895). https://www.bmj.com/content/346/bmj.f324. 10.1136/bmj.f324 PMC3558410 23360717

[40] Levey AS, Coresh J, Tighiouart H, Greene T, Inker LA, Measured. and estimated glomerular filtration rate: current status and future directions [Internet]. Vol. 16, Nature Reviews Nephrology. Nature Publishing Group; 2020 [cited 2022 Jul 15]. pp. 51–64. https://www.nature.com/articles/s41581-019-0191-y. 10.1038/s41581-019-0191-y 31527790

[41] Stengel B, Metzger M, Froissart M, Rainfray M, Berr C, Tzourio C et al. Epidemiology and prognostic significance of chronic kidney disease in the elderly–the Three-City prospective cohort study. Nephrol Dial Transplant [Internet]. 2011 [cited 2022 Jul 15];26(10):3286–95. https://pubmed.ncbi.nlm.nih.gov/21677301/. 10.1093/ndt/gfr323 PMC3925095 21677301

[42] Batte A, Berrens Z, Murphy K, Mufumba I, Sarangam ML, Hawkes MT (2021). Malaria-associated acute kidney injury in African children: prevalence, pathophysiology, impact, and management challenges. Int J Nephrol Renovasc Dis.

[43] Faraj J, Mander J, Burnett JR, Prentice D (2017). Filiarial chyluria with nephrotic-range proteinuria and associated hypoalbuminaemia and hypogammaglobulinaemia secondary to bilateral lymphorenal fistulae. BMJ Case Rep.

[44] van Velthuysen MLF, Florquin S (2000). Glomerulopathy Associated with parasitic infections. Clin Microbiol Rev.

[45] Daher EDF, da Silva Junior GB, Trivedi M, Fayad T, Srisawat N, Nair S et al. Kidney complications of parasitic diseases [Internet]. Vol. 18, Nature Reviews Nephrology. Nature Publishing Group; 2022 [cited 2022 Jul 15]. pp. 396–406. https://www.nature.com/articles/s41581-022-00558-z. 10.1038/s41581-022-00558-z 35347315

[46] Burchard GD, Kubica T, Tischendorf FW, Kruppa T, Brattig NW. Analysis of renal function in onchocerciasis patients before and after therapy. Am J Trop Med Hyg [Internet]. 1999 [cited 2022 Jul 15];60(6):980–6. https://www.academia.edu/download/42282386/980.pdf. 10.4269/ajtmh.1999.60.980 10403331

[47] Ngu JL, Chatelanat F, Leke R, Ndumbe P, Youmbissi J. Nephropathy in Cameroon: Evidence for filarial derived immune-complex pathogenesis in some cases. Clin Nephrol [Internet]. 1985 [cited 2022 Jul 15];24(3):128–34. https://europepmc.org/article/med/3862488. 3862488

[48] Njim T, Aminde LN. An appraisal of the neglected tropical diseases control program in Cameroon: the case of the national program against onchocerciasis. BMC Public Health. 2017;17(1). 10.1186/s12889-017-4037-x PMC5251311 28109269

[49] Dana D, Debalke S, Mekonnen Z, Kassahun W, Suleman S, Getahun K et al. A community-based cross-sectional study of the epidemiology of onchocerciasis in unmapped villages for community directed treatment with ivermectin in Jimma Zone, southwestern Ethiopia. BMC Public Health. 2015;15(1). 10.1186/s12889-015-1888-x PMC4486700 26130117

[50] Crump A, Morel CM, Omura S. The onchocerciasis chronicle: From the beginning to the end? [Internet]. Vol. 28, Trends in Parasitology. 2012 [cited 2022 Jul 15]. pp. 280–8. 10.1016/j.pt.2012.04.005. 10.1016/j.pt.2012.04.005 22633470

[51] Kifle B, Woldemichael K, Nigatu M (2019). Prevalence of Onchocerciasis and Associated Factors among adults aged ≥ 15 years in Semen Bench District, Bench Maji Zone, Southwest Ethiopia: community based cross-sectional study. Adv Public Heal.

[52] Biritwum NK, de Souza DK, Asiedu O, Marfo B, Amazigo UV, Gyapong JO. Onchocerciasis control in Ghana (1974–2016). Parasites and Vectors [Internet]. 2021 Dec 1 [cited 2021 Dec 14];14(1):1–9. https://parasitesandvectors.biomedcentral.com/articles/10.1186/s13071-020-04507-2. 10.1186/s13071-020-04507-2 PMC7778817 33388081

[53] Gutiérrez-Peña EJ, Knab J, Büttner DW. Neutrophil granule proteins: Evidence for the participation in the host reaction to skin *microfilariae* of Onchocerca volvulus after diethylcarbamazine administration. Parasitology [Internet]. 1996 [cited 2022 Jan 7];113(4):403–14. https://www.cambridge.org/core/journals/parasitology/article/neutrophil-granule-proteins-evidence-for-the-participation-in-the-host-reaction-to-skin-microfilariae-of-onchocerca-volvulus-after-diethylcarbamazine-administration/11CDA0D4CBF4CDF8D2A4B8DD2988A. 10.1017/s0031182000066543 8873478

[54] Darge K, Lucius R, Monson MH, Behrendsen J, Buttner DW. Immunohistological and electron microscopic studies of microfilariae in skin and lymph nodes from onchocerciasis patients after ivermectin treatment. Trop Med Parasitol [Internet]. 1991 [cited 2022 Jan 7];42(4):361–7. https://europepmc.org/article/med/1796234. 1796234

[55] Cooper PJ, Awadzi K, Ottesen EA, Remick D, Nutman TB. Eosinophil sequestration and activation are associated with the onset and severity of systemic adverse reactions following the treatment of onchocerciasis with ivermectin. J Infect Dis [Internet]. 1999 Mar 1 [cited 2021 Nov 12];179(3):738–42. https://academic.oup.com/jid/article/179/3/738/809600. 10.1086/314647 9952390

[56] Wildenburg G, Darge K, Knab J, Tischendorf FW, Bonow I, Buttner DW. Lymph nodes of onchocerciasis patients after treatment with ivermectin: Reaction of eosinophil granulocytes and their cationic granule proteins. Trop Med Parasitol [Internet]. 1994 Jun 1 [cited 2021 Nov 12];45(2):87–96. https://europepmc.org/article/med/7939167. 7939167

[57] Folkard SG, Hogarth PJ, Taylor MJ, Bianco AE. Eosinophils are the major effector cells of immunity to microfilariae in a mouse model of onchocerciasis. Parasitology [Internet]. 1996 [cited 2021 Nov 12];112(3):323–9. https://www.cambridge.org/core/journals/parasitology/article/abs/eosinophils-are-the-major-effector-cells-of-immunity-to-microfilariae-in-a-mouse-model-of-onchocerciasis/917290D05CA62F2BC62FC43C9E82DD85. 10.1017/s0031182000065847 8728996

[58] Wildenburg G, Krömer M, Büttner DW. Dependence of eosinophil granulocyte infiltration into nodules on the presence of microfilariae producing Onchocerca volvulus. Parasitol Res [Internet]. 1996 [cited 2022 Jan 7];82(2):117–24. https://link.springer.com/article/10.1007/s004360050081. 10.1007/s004360050081 8825205

[59] Nfon CK, Makepeace BL, Njongmeta LM, Tanya VN, Bain O, Trees AJ (2006). Eosinophils contribute to killing of adult Onchocerca ochengi within onchocercomata following elimination of Wolbachia. Microbes Infect.

[60] Obata-Ninomiya K, Domeier PP, Ziegler SF. Basophils and eosinophils in Nematode infections. Frontiers in Immunology. Volume 11. Frontiers Media S.A.; 2020. p. 3014. 10.3389/fimmu.2020.583824 PMC7737499 33335529

[61] Ohnmacht C, Voehringer D (2009). Basophil effector function and homeostasis during helminth infection. Blood.

[62] Torrero MN, Hübner MP, Larson D, Karasuyama H, Mitre E. Basophils Amplify Type 2 Immune Responses, but Do Not Serve a Protective Role, during Chronic Infection of Mice with the Filarial Nematode Litomosoides sigmodontis. J Immunol [Internet]. 2010 Dec 15 [cited 2022 Jan 5];185(12):7426–34. https://pubmed.ncbi.nlm.nih.gov/21057084/. 10.4049/jimmunol.0903864 21057084

[63] Uciechowski P, Rink L, Basophil. Eosinophil, and Neutrophil Functions in the Elderly. Immunol Aging [Internet]. 2014 Jan 1 [cited 2022 Jan 5];47–63. https://link.springer.com/chapter/10.1007/978-3-642-39495-9_5.

[64] Brattig NW, Büttner DW, Hoerauf A. Neutrophil accumulation around Onchocerca worms and chemotaxis of neutrophils are dependent on Wolbachia endobacteria. Microbes Infect [Internet]. 2001 [cited 2022 Jan 7];3(6):439–46. https://www.sciencedirect.com/science/article/pii/S1286457901013995. 10.1016/s1286-4579(01)01399-5 11377205

[65] Pionnier N, Brotin E, Karadjian G, Hemon P, Gaudin-Nomé F, Vallarino-Lhermitte N et al. Neutropenic Mice Provide Insight into the Role of Skin-Infiltrating Neutrophils in the Host Protective Immunity against Filarial Infective Larvae. PLoS Negl Trop Dis [Internet]. 2016 Apr 25 [cited 2022 Jan 7];10(4):e0004605. https://journals.plos.org/plosntds/article?id=10.1371/journal.pntd.0004605. 10.1371/journal.pntd.0004605 PMC4844152 27111140

[66] Tamarozzi F, Turner JD, Pionnier N, Midgley A, Guimaraes AF, Johnston KL et al. Wolbachia endosymbionts induce neutrophil extracellular trap formation in human onchocerciasis. Sci Rep [Internet]. 2016 Oct 18 [cited 2022 Jan 5];6(1):1–13. https://www.nature.com/articles/srep35559. 10.1038/srep35559 PMC5067710 27752109

[67] Njoo FL, Hack CE, Oosting J, Stilma JS, Kijlstra A. Neutrophil activation in ivermectin-treated onchocerciasis patients. Clin Exp Immunol [Internet]. 1993 Nov 1 [cited 2022 Jan 5];94(2):330–3. https://pubmed.ncbi.nlm.nih.gov/8222324/. 10.1111/j.1365-2249.1993.tb03452.x PMC1534235 8222324

[68] Francis H, Awadzi K, Ottesen EA. The Mazzotti reaction following treatment of onchocerciasis with diethylcarbamazine: Clinical severity as a function of infection intensity. Am J Trop Med Hyg [Internet]. 1985 [cited 2022 Jul 19];34(3):529–36. https://europepmc.org/article/med/4003668. 10.4269/ajtmh.1985.34.529 4003668

[69] Keiser PB, Reynolds SM, Awadzi K, Ottesen EA, Taylor MJ, Nutman TB. Bacterial endosymbionts of Onchocerca volvulus in the pathogenesis of posttreatment reactions. J Infect Dis [Internet]. 2002 [cited 2022 Jul 19];185(6):805–11. https://academic.oup.com/jid/article-abstract/185/6/805/878640. 10.1086/339344 11920298

[70] Sulaiman WAW, Kamtchum-Tatuene J, Mohamed MH, Ramachandran V, Ching SM, Lim SMS et al. Anti-Wolbachia therapy for onchocerciasis & lymphatic filariasis: Current perspectives [Internet]. Vol. 149, Indian Journal of Medical Research. Wolters Kluwer Medknow Publications; 2019 [cited 2022 Jul 19]. pp. 706–14. https://journals.lww.com/ijmr/Fulltext/2019/49060/Anti_Wolbachia_therapy_for_onchocerciasis__.4.aspx. 10.4103/ijmr.IJMR_454_17 PMC6755775 31496523

[71] Brescia G, Barion U, Zanotti C, Cinetto F, Giacomelli L, Martini A et al. Blood eosinophil-to-basophil ratio in patients with sinonasal polyps: Does it have a clinical role? Ann Allergy Asthma Immunol [Internet]. 2017 Sep 1 [cited 2022 Jan 6];119(3):223–6. https://pubmed.ncbi.nlm.nih.gov/28743424/. 10.1016/j.anai.2017.06.008 28743424

[72] Mirna M, Schmutzler L, Topf A, Hoppe UC, Lichtenauer M. Neutrophil-to-lymphocyte ratio and monocyte-to-lymphocyte ratio predict length of hospital stay in myocarditis. Sci Rep [Internet]. 2021 Sep 13 [cited 2022 Jan 6];11(1):1–9. https://www.nature.com/articles/s41598-021-97678-6. 10.1038/s41598-021-97678-6 PMC8438016 34518607

[73] Unnasch TR, Golden A, Cama V, Cantey PT. Diagnostics for onchocerciasis in the era of elimination [Internet]. Vol. 10, International Health. Oxford University Press; 2018 [cited 2022 Jul 19]. pp. i20–6. /pmc/articles/PMC5881263/. 10.1093/inthealth/ihx047 PMC5881263 29471336

[74] Üstündağ Y, Kazanci G, Koloğlu E, Çağlak RF, Yildirim HA, Arikan FY. E, A retrospective study of age-defined hematologic inflammatory markers related to pediatric COVID-19 diagnosis. Int J Lab Hematol [Internet]. 2022 Aug 1 [cited 2022 Jul 19];44(4):722–8. https://onlinelibrary.wiley.com/doi/full/10.1111/ijlh.13838. 10.1111/ijlh.13838 PMC9111715 35437914

[75] Kant A, Terzioğlu K. Association of Severity of Allergic Rhinitis With Neutrophil-To-Lymphocyte, Eosinophil-To-Neutrophil, And Eosinophil-To-Lymphocyte Ratios In Adults. Allergol Immunopathol (Madr) [Internet]. 2021 Sep 1 [cited 2022 Jan 6];49(5):94–9. https://all-imm.com/index.php/aei/article/view/204/606. 10.15586/aei.v49i5.204 34476928

[76] Adu Mensah D, Debrah LB, Gyamfi PA, Rahamani AA, Opoku VS, Boateng J, Obeng P, Osei-Mensah J, Kroidl I, Klarmann-Schulz U, Hoerauf A, Debrah AY. Occurrence of Lymphatic Filariasis infection after 15 years of mass drug administration in two hotspot districts in the Upper East Region of Ghana. PLoS Negl Trop Dis. 2022;16(8):e0010129. 10.1371/journal.pntd.0010129 PMID: 35926012. 10.1371/journal.pntd.0010129 PMC9380951 35926012

[77] Phillips RO, Frimpong M, Sarfo FS, Kretschmer B, Beissner M, Debrah A, Ampem-Amoako Y, Abass KM, Thompson W, Duah MS, Abotsi J (2014). Infection with Mansonella perstans nematodes in Buruli ulcer patients, Ghana. Emerg Infect Dis.

[78] Debrah LB, Nausch N, Opoku VS, Owusu W, Mubarik Y, Berko DA, Wanji S, Layland LE, Hoerauf A, Jacobsen M, Debrah AY (2017). Epidemiology of Mansonella perstans in the middle belt of Ghana. Parasites Vectors.

